# Exploring specific biomarkers in blood for *in vitro* diagnosis of caprine tuberculosis

**DOI:** 10.3389/fmicb.2026.1765857

**Published:** 2026-02-20

**Authors:** Carlos Velasco, Alvaro Roy, Fatima Cruz-Lopez, Alberto Gomez-Buendia, Javier Ortega, Sofía Mendez-Lopez, Lucia de Juan, Lucas Dominguez, Beatriz Romero, Julio Alvarez, Javier Bezos

**Affiliations:** 1 VISAVET Health Surveillance Centre, Complutense University of Madrid, Madrid, Spain; 2 Departamento de Sanidad Animal, Facultad de Veterinaria, Universidad Complutense de Madrid, Madrid, Spain; 3 Centro Nacional de Epidemiología, Instituto de Salud Carlos III, Madrid, Spain

**Keywords:** acute phase proteins, complete blood count, diagnosis, goat, IFN-γ, IP-10, oxidative stress, tuberculosis

## Abstract

**Introduction:**

Caprine tuberculosis (cTB) is a zoonotic disease with significant implications for animal and public health and economic impact. Therefore, accurate ante-mortem diagnosis is essential. Diagnosis in goats rely on the single and comparative intradermal tuberculin test (SITT and CITT, respectively), both of which showing limited diagnostic performance. The aim of the study was to characterize the immune and physiological responses to cTB in naturally infected goats and to identify potential *in vitro* biomarkers that could improve the reliability of cTB diagnosis.

**Methodology:**

The study was conducted in a cTB infected herd and goats were classified as reactors (positive to SITT or IFN-γ release assay and considered as infected) and non-reactors. Basal production of fifteen key immune cytokines was measured in plasma samples (*n* = 19) and compared with levels observed after stimulation with bovine purified protein derivative (PPDb). Acute phase proteins (haptoglobin/Hp and serum amyloid-A/SAA) and oxidative stress markers (total antioxidant status/TAS and malondialdehyde/MDA) were determined in serum samples (*n* = 90) while complete blood count (CBC) was performed on whole-blood samples (*n* = 44).

**Results and discussion:**

Regarding cytokine expression patterns, plasma levels of IFN-γ and IFN-γ-inducible protein 10 (IP-10) in PPDb-stimulated samples were significantly higher (*p* < 0.005 and *p* < 0.001, respectively) in reactor goats than in non-reactor. When considering the difference between PPDb and PBS-stimulated samples (Δ-cytokine), levels of Δ-IFN-γ, Δ-IP-10 and Δ-IL-1α were significantly higher (*p* = 0.0006, *p* < 0.0001 and *p* = 0.022, respectively) in reactor goats compared to non-reactor. In addition, significantly higher (*p* = 0.014) levels of serum TAS were observed in reactor vs. non-reactor goats whereas no significant differences (*p* > 0.05) were found for Hp, SAA and MDA. Finally, among the CBC parameters, only the percentage of mid-range cells was significantly higher (*p* < 0.05) in reactor vs. non-reactor goats. This study demonstrates the differential expression of immunological (IFN-γ, IP-10 and IL-1α) and physiological (TAS) biomarkers in goats reacting to TB diagnostic tests and suggest their potential as biomarkers of cTB infection. These findings could lead to the development of novel diagnostic *in vitro* methodologies and contribute to more effective cTB eradication strategies.

## 1 Introduction

Caprine tuberculosis (cTB) is an infectious worldwide-distributed disease caused by mycobacteria of the *Mycobacterium tuberculosis* complex (MTBC) including *M. bovis* and *M. caprae* (Aranaz et al., 2003; Malone and Gordon, 2017), both representing a significant concern for animal and public health due to their zoonotic potential (Pérez De Val et al., 2025; Martínez-Lirola et al., 2023). In Spain, the country that hosts the second largest population of goats in the European Union (2.3 million in 2024) (Eurostat, 2025), cTB control is achieved through regional eradication programs based on the cull of positive animals to the single or comparative intradermal tuberculin test (SITT or CITT, respectively) along with slaughterhouse surveillance (Bezos et al., 2014b; Roy et al., 2020). However, tuberculin tests present some disadvantages such as the subjective component in test interpretation, imperfect test accuracy and the need of handling the animal twice (one for testing and one or reading the results) (Bezos et al., 2014b; De La Rua-Domenech et al., 2006). Hence, the development of new *in vitro* methodologies that can overcome these limitations of tuberculin tests and contribute to improving the accuracy of TB diagnosis in this species would be highly advantageous.

In the early stages of infection, the tuberculous mycobacteria typically colonize the lungs (Seva et al., 2000; Domingo et al., 2014; de Val et al., 2011), where the alveolar macrophages play a pivotal role in recognizing and phagocytosing the mycobacteria (Palmer et al., 2022; Agulló-Ros et al., 2025). This leads to the activation of the host immune response and the production of pro-inflammatory cytokines including interferon-gamma (IFN-γ) (Etna et al., 2014; Schiller et al., 2009; Palmer et al., 2020). In fact, IFN-γ detection through the whole blood-based IFN-γ release assay (IGRA) is used as an official test in bovine TB (bTB) eradication programs within the European Union. This test relies on the assessment of the levels of secretion of this cytokine by sensitized T-cells after *in vitro* stimulation with bovine and avian purified protein derivatives (PPDb and PPDa, respectively) (Schiller et al., 2009; Bezos et al., 2014a). Beyond IFN-γ, other cytokines play a crucial role in the host’s immune response against MTBC, highlighting their potential as immunological biomarkers for TB infection (Smith et al., 2021a,b; Franzoni et al., 2024). Previous studies have demonstrated that the detection of IFN-γ-inducible protein 10 (IP-10) could maximize the detection of TB in cattle (Coad et al., 2019; Steinbach et al., 2021; Parsons et al., 2016), African buffalo (Goosen et al., 2015; Bernitz et al., 2019), water buffalo (Franzoni et al., 2024) and goats (Cooke et al., 2023). However, the pattern expression of these and other cytokines in goats exposed to MTBC has only been evaluated in serum samples (Ortega et al., 2022) but not after specific stimulation of plasma samples with PPDb.

Simultaneously to cytokine production, MTBC infection triggers the production of acute phase proteins (APPs) (Vicente et al., 2019; Lowie et al., 2025) and reactive oxygen and nitrogen species (ROS and RNS, respectively) (Palanisamy et al., 2011; Gassó et al., 2016) as part of the host non-specific innate immune response against the mycobacteria. APPs are liver-derived serum proteins in response to pro-inflammatory cytokines (mainly IL-1β, IL-6, and TNF-α) (Saco and Bassols, 2023; Murata et al., 2004; Iliev and Georgieva, 2018) to enhance the immune and inflammatory response (Eckersall, 2004; Eckersall and Bell, 2010), with haptoglobin (Hp) and serum amyloid-A (SAA) as the major APPs in goats (Iliev and Georgieva, 2018; González et al., 2008). Hp binds free hemoglobin and reduces available iron for the pathogens targeted (Petersen et al., 2004) while SAA causes bacteria opsonization (Hari-Dass et al., 2005) and promote chemotactic recruitment of inflammatory cells to sites of lesion (Petersen et al., 2004). ROS and RNS are produced during MTBC infection by macrophages and neutrophils aimed at mycobacterial clearance (Palanisamy et al., 2011; Gassó et al., 2016; Shastri et al., 2018) but can collaterally cause host-tissue injury and physiological disorders, known as oxidative stress (OS). Thus, OS results from the imbalance between the ROS/RNS production and the host-ability to absorb their harmful effects through antioxidant agents (Blanca et al., 2024; Al-Hassan, 2018). Several biomarkers can be quantified for OS assessment including antioxidant agents, ROS or RNS (Gassó et al., 2016; Blanca et al., 2024; Pellegrini et al., 2020). However, ROS/RNS have an extremely short half-life and are difficult to measure directly (Gassó et al., 2016). In this sense, OS can be assessed by measuring products of ROS/RNS-mediated modifications such as the malondialdehyde (MDA), a metabolite from lipid peroxidation (Al-Hassan, 2018; Perez-Montero et al., 2025; Turk et al., 2008). Furthermore, the total antioxidant status (TAS) provides a comprehensive measure of the antioxidant potential of a sample with fewer logistical requirements than analyzing individual antioxidants (Beghelli et al., 2016). However, there is a lack of knowledge regarding APPs expression and OS response against MTBC infection. Moreover, even though the complete blood count (CBC) parameters could be of interest in the diagnosis and prognosis of TB as shown in humans (Shah et al., 2022; Balepur and Schlossberg, 2016), very few studies—none in goats—have addressed the variations in CBC associated with MTBC infection in animals (Thapa et al., 2021; Miller et al., 1989).

Thus, in this study we aimed to characterize the immune and physiological response against MTBC and to screen for potential biomarkers related with the reactivity to TB diagnostic techniques. Specifically, we evaluated (i) the pattern expression of a panel of 15 key immune cytokines in plasma samples stimulated with PBS or PPDb, (ii) the APPs and OS response in serum samples and (iii) the CBC under field conditions in goats from a TB infected herd.

## 2 Materials and methods

### 2.1 Animals and study design

The study was carried out in a dairy herd of mixed-breed goats located in Castilla La-Mancha (central region of Spain) in the framework of the regional mandatory cTB eradication program (DOCM 2023/617). Six months before the study, the herd was confirmed to be infected by bacteriological culture (*M. bovis;* SB2232) and had an apparent prevalence of 20.8% based on routine CITT testing. A total of ninety goats (*n* = 90) were randomly selected and enrolled in the study. At day 0, all goats were subjected to SITT, IGRA and serum samples were collected for APPs (Hp and SAA) and OS (TAS and MDA) determination. Also at this day, whole blood samples from forty-four randomly selected goats were obtained for CBC and plasma cytokines/chemokines collected for the IGRA were quantified from nineteen goats randomly selected. Sample size was determined by logistical constraints and by the total of analyses per commercial kit.

### 2.2 Single intradermal tuberculin test

SITT involved the intradermal inoculation of 0.1 mL of PPDb (CZ Vaccines, Porriño, Spain) on the left side of the neck using a Dermojet syringe (Akra Dermojet, Pau, France). An animal was classified as reactor if the skin fold thickness increased by ≥ 4 mm and/or if clinical signs such as oedema, pain, exudation, or necrosis were observed. SITT was conducted in accordance with Regulation EU 2016/429, Commission Delegated Regulation EU 2020/688, Spanish Royal Decree 2611/1996, and the standard operating procedures (SOPs) of the European Union Reference Laboratory (EU-RL) for bTB for intradermal tuberculin testing in caprine animals (SOP/002/EURL).

### 2.3 Whole blood stimulation and interferon gamma release assay

Blood samples were collected by venipuncture using evacuated tubes with lithium heparin (BD Vacutainer Becton, Dickinson and Company, Franklin Lakes, United States). Then, blood samples were stimulated in the laboratory with PBS (as a non-stimulating control) and PPDb and PPDa (CZ Vaccines, Porriño, Spain) at a final concentration of 20 μg/mL in order to evaluate IFN-γ production (SOP/004/EURL). Afterwards, plasma was obtained and IFN-γ production was measured using a commercial IGRA kit (Bovigam TB, ThermoFisher Scientific, Waltham, United States) according to the manufacturer’s instructions (Gomez-Buendia et al., 2023b). Briefly, an animal was considered as IGRA-reactor if the optical density (OD) of a sample stimulated with PPDb minus the OD of PBS was greater than 0.1 and greater than the OD of the sample stimulated with PPDa.

### 2.4 MILLIPLEX^®^ bovine cytokine/chemokine magnetic bead panel 1

A 15-plex Luminex-based cytokine/chemokine panel was applied to plasma samples collected at day 0 of the study and stimulated with PBS or PPDb. Levels of IFN-γ, IL-1α, IL-1β, IL-4, IL-6, IL-8, IL-10, IL-17A, IL-36RA, IP-10, MCP-1, MIP-1α MIP-1β, TNF-α and VEGF-A were measured using a bovine multiplex assay kit (MILLIPLEX^®^ Bovine Cytokine/Chemokine Magnetic Bead Panel 1, 96-Well Plate Assay, Merck Millipore, United Kingdom) according to manufacturer’s instructions. Briefly, plasma samples were centrifuged at 10,000 g for 5 min and diluted 1:2 in the buffer provided in the kit. Twenty-five μL of each mix (plasma from whole blood stimulated with PBS or PPDb and buffer) was then analyzed in duplicate on Bio-Plex 200 System (Bio-Rad, Hercules, CA, United States). Then, levels of each cytokine were calculated by the mean of the two analyses, multiplied by the dilution factor and expressed in pg/mL.

### 2.5 Acute phase proteins

Blood samples were collected by venipuncture using evacuated tubes with no additives (BD Vacutainer Becton, Dickinson and Company, Franklin Lakes, United States). The samples were then centrifuged at 1,500 g for 10 min to obtain serum, which was stored at −80 °C until analysis. Serum concentrations of Hp (mg/mL) in undiluted samples were quantified using a commercial kit (PHASE Haptoblogin Assay, Tridelta, Maynooth, Ireland). The Hp assay is based on a colorimetric method that detects the peroxidase activity of the haptoglobin-hemoglobin complex. SAA concentration (mg/L) was measured using a sandwich-type ELISA commercial kit (PHASE SAA Assay, Tridelta, Maynooth, Ireland) according to manufacturer’s instructions but using a lower dilution (1:10) of samples.

### 2.6 Oxidative stress response

Serum TAS was determined using a TAS-liquid stable colorimetric kit (Fortress Diagnostics Limited, Belfast, United Kingdom) based on the ABTS assay. This methodology relies on the reaction between ABTS, a peroxidase (metmyoglobin) and hydrogen peroxide, resulting in a color change of the sample that is determined spectrophotometrically at 660 nm. The suppression of color change is proportional to the concentration of antioxidants in the sample. Results were expressed in mmol Trolox Equivalent/L and indicates the amount of antioxidants in the sample relative to the Trolox standard (Perez-Montero et al., 2024; Erel, 2004). MDA was determined through a Thiobarbituric Acid Reactive Substances (TBARS) assay (Cayman Chemicals, United States). This method is based on the reaction between MDA and TBARS under high-temperatures and acid conditions, leading to the formation of an MDA-TBARS adduct. Then, the MDA-TBARS adduct is measured by spectrophotometry at 540 nm. The concentration of MDA was calculated and expressed in micromolar (μM) units (Perez-Montero et al., 2025; Aguilar Diaz De Leon and Borges, 2020).

### 2.7 Complete blood count

Blood samples were drawn from the jugular vein using evacuated tubes with K_3_ EDTA (BD Vacutainer Becton, Dickinson and Company, Franklin Lakes, United States) and analyzed with an auto hematology analyzer (Dymind DH36, Dymind Biotechnology Co., Shenzhen, China). The following variables were determined: total white blood cell (WBC; x10^3^/μL), total (x10^3^/μL) and percentage (%) of lymphocytes (Lym), granulocytes (Gran) and mid-range cells (Mid), total red blood cell count (RBC; x10^6^/μL), packed cell volume (PCV; %), hemoglobin concentration (HGB; g/dL), mean cell volume (MCV; fL), mean cell hemoglobin (MCH; pg), mean corpuscular hemoglobin concentration (MCHC; g/dL), standard deviation and coefficient of variation of red cell distribution width (RDW-SD and RDW-CV, respectively), total platelets (PLT; 10^3^/μL), mean platelet volume (MPV; fL), platelet distribution width (PDW) and plateletcrit (PCT; %).

### 2.8 Statistical analysis

Goats were divided into two groups according to their reactivity to the TB diagnostic tests: reactor goats (with a positive result to SITT or IGRA and considered infected) and non-reactors (Table 1). Quantitative differences in the levels of plasma cytokines in samples stimulated with PBS vs. PPDb (reagent effect) within non-reactor and reactor groups were assessed using a Wilcoxon-signed rank test. Quantitative differences in the levels of the 15 cytokines in plasma stimulated with PBS or PPDb as well as the difference between them (Δ-cytokine, calculated as the difference between the cytokine level after PPDb stimulation minus its paired PBS) in non-reactor vs. reactor goats (group effect) were evaluated using a Mann–Whitney U test. For both comparisons, *p*-values were adjusted using the Bonferroni correction to control for multiple testing. Quantitative differences in the levels of Hp, SAA, MDA, TAS and CBC parameters between non-reactor and reactor goats were analyzed using the Mann–Whitney U test. Spearman’s rank correlation coefficient (r_s_) was used to assess the relationship between the following pairs of variables: (i) OD values of IFN-γ from the Bovigam and plasma levels of this cytokine obtained with the MILLIPEX in samples stimulated with PBS or PPDb, (ii) the concentrations of plasma IFN-γ and IP-10 in samples stimulated with PBS or PPDb as well as in Δ-cytokine levels, (iii) the levels of baseline plasma pro-inflammatory cytokines (IL-1β, IL-6 and TNF-α) and serum APPs (Hp and SAA), (iv) the concentration of serum Hp and SAA and (v) the levels of serum TAS and MDA. Data visualization and statistical analyses were conducted using R Studio version 4.2.1 (R Core Team, 2025) and results were interpreted using a significance threshold of *p* < 0.05.

**Table 1:** Techniques used and number and infection status of the goats tested in the study.

Technique	Reactor^a^		Non-reactor^b^		Total
	*n*	%	*n*	%	*n*
MILLIPLEX^®^ bovine cytokine/chemokine magnetic bead panel 1	10	52.6	9	47.4	19
PHASE Hp assay	34	37.8	56	62.2	90
PHASE SAA assay	34	37.8	56	62.2	90
TAS kit	34	37.8	56	62.2	90
TBARS assay kit	34	37.8	56	62.2	90
CBC	21	47.8	23	52.2	44

a Goats with a positive result to single intradermal tuberculin test or IFN-γ release assay.

b Goats with a negative result to single intradermal tuberculin test and IFN-γ release assay.

## 3 Results

**Figure 1:**
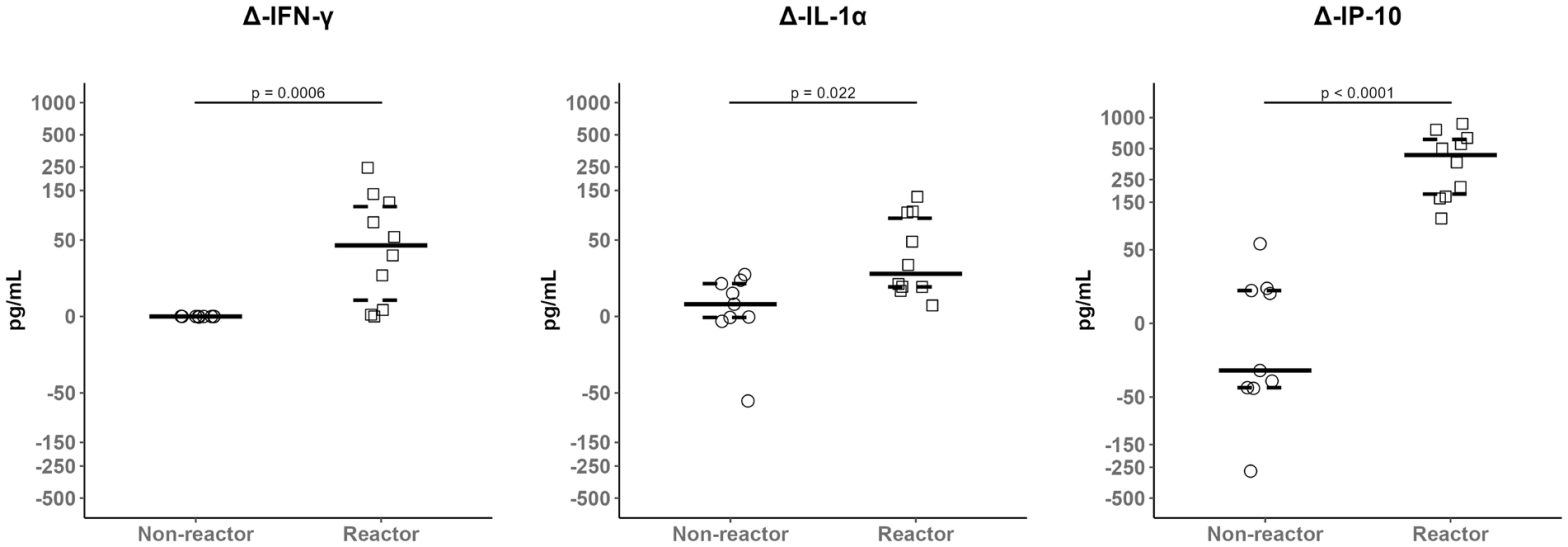
Δ-IFN-γ, Δ-IL-1α, and Δ-IP-10 plasma levels (pg/mL) in non-reactor (single intradermal tuberculin test/SITT and IFN-γ release assay/IGRA negatives; *n* = 9) and reactor goats (SITT or IGRA positives; *n* = 10). Solid line represents the median and dashed lines represent the 25th (lower) and 75th (upper) percentiles.

**Figure 2:**
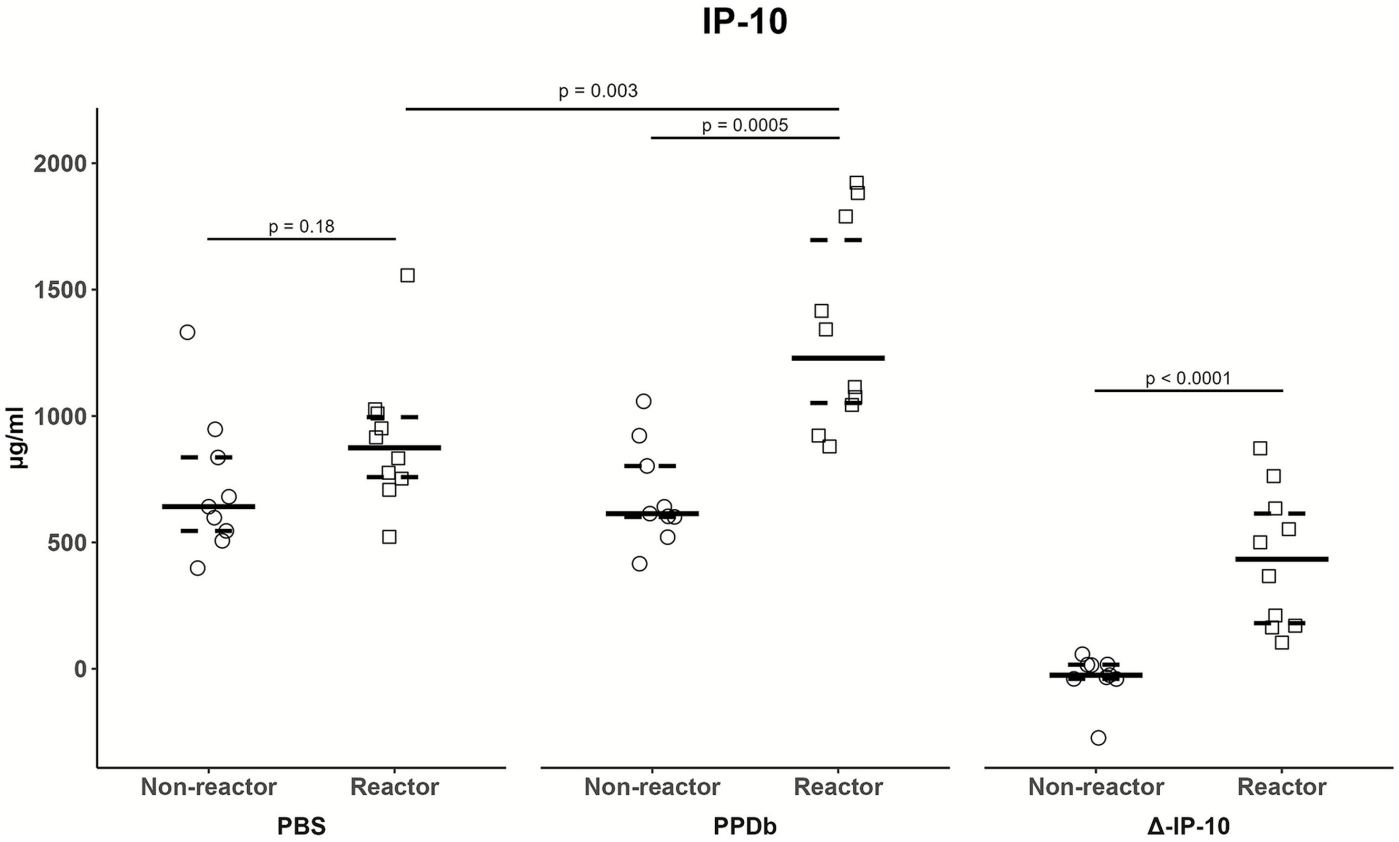
Distribution of baseline, PPDb-stimulated and Δ-IP-10 plasma levels (pg/mL) in non-reactor (single intradermal tuberculin test/SITT and IFN-γ release assay/IGRA negatives; *n* = 9) and reactor goats (SITT or IGRA positives; *n* = 10). Solid line represents the median and dashed lines represent the 25th (lower) and 75th (upper) percentiles.

Of the fifteen cytokines evaluated using the Luminex-based panel, significant differences in plasma levels in whole blood samples stimulated with PPDb compared to PBS (reagent effect) in reactor goats were found for the IFN-γ, IL-1α, IL-6, IL-8, IL-10, MIP-1α, IL-36RA, IP-10 and VEGF-A (Table 2). However, only plasma levels of IFN-γ [*p =* 0.0019; median difference (MD) = 45.1] and IP-10 (*p* = 0.0005; MD = 614.7) after PPDb stimulation in reactor goats were significantly higher than those found in the non-reactors (group effect). When considering the difference PPDb-PBS, significant differences in the levels of Δ-IFN-γ (*p* = 0.0006; MD = 44.0), Δ-IP-10 (*p* < 0.0001; MD = 458.8) and Δ-IL-1α (*p* = 0.022; MD = 15.7) in reactor *vs*. non-reactors were found (Table 2; Figures 1, 2). In addition, levels of plasma IP-10 regardless the reagent used for whole blood stimulation, as well as Δ-IP-10 concentrations, exceeded those respective of IFN-γ and IL-1α in reactor goats (Supplementary Table 1). A strong correlation (r_s_ = 0.73; *p* = 0.0003) was observed between OD values of IFN-γ from the Bovigam kit and IFN-γ levels obtained with the MILLIPEX in plasma samples stimulated with PPDb, whereas no correlation (r_s_ = 0.38; *p* > 0.05) was observed between these variables in non-stimulated samples. Moreover, a strong correlation (r_s_ = 0.64; *p* = 0.003) was observed between IFN-γ and IP-10 baseline levels, while this relationship was stronger after PPDb stimulation (r_s_ = 0.80; *p* < 0.0001) or when considering the Δ-cytokine level (r_s_ = 0.76; *p* < 0.001).

**Table 2:** Differences (*p*-values and median differences/MD) on cytokine levels between reactor (single intradermal tuberculin test/SITT or IFN-γ release assay/IGRA positives; *n* = 10) vs. non-reactor (SITT and IGRA negatives; *n* = 9) goats (group effect) or between reagents used for whole-blood stimulation (PPDb vs. PBS; reagent effect).

Cytokine^a^	Group effect						Reagent effect			
	PBS		PPDb		Δ-cytokine^a^		Reactor		Non-reactor	
	*p-*value^b^	MD	*p-*value^b^	MD	*p-*value^b^	MD	*p-*value^c^	MD	*p-*value^c^	MD
IFN-γ	0.053	0.2	**0.0019**	45.1	**0.0006**	44.0	**0.018**	44.9	1	0.0
IL-1α	1	4.9	0.18	28.2	**0.022**	15.7	**0.003**	31.7	0.8	8.5
IL-1β	0.6	0.0	0.6	0.0	0.3	0.0	1	0.0	1	0.0
IL-4	1	−1.3	0.8	−6.8	0.6	−4.6	1	−2.1	0.8	3.3
IL-6	1	−5.3	0.4	55.4	0.13	107.2	**0.007**	460.4	0.2	399.6
IL-8	1	−185.2	1	223.6	0.2	189.7	**0.003**	497.6	0.16	88.6
IL-10	0.8	23.4	0.3	33.1	0.18	16.3	**0.003**	47.4	0.2	37.8
IL-17A	0.4	−0.3	1	0.09	0.6	0.0	0.072	0.3	0.2	0.1
MIP-1α	1	−206.2	1	120.2	0.3	126.6	**0.003**	474.1	**0.015**	147.7
IL-36RA	1	10.1	1	7.4	0.3	−7.0	**0.011**	−20.0	0.6	−17.3
IP-10	0.18	232.9	**0.0005**	614.7	**<0.0001**	458.8	**0.003**	354.4	0.7	−27.3
MCP-1	0.7	3.7	0.18	51.7	0.7	21.9	0.6	28.8	1	−26.7
MIP-1β	0.7	0.0	0.7	0.0	0.3	0.0	1	0.0	1	0.0
TNF-α	0.6	1095.4	0.6	961.5	0.7	−57.4	0.9	−124.6	1	9.2
VEGF-A	0.5	−34.5	0.2	−111.2	0.053	−20.4	**0.003**	−76.0	**0.023**	0.6

*p*-values <0.05 are marked in bold.

a Δ-cytokine was calculated as the difference between the cytokine level under PPDb stimulation and its paired PBS baseline.

b Mann–Whitney U test.

c Wilcoxon-signed rank test.

**Figure 3:**
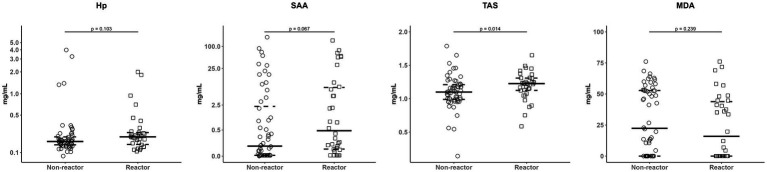
Serum levels of haptoglobin (Hp; mg/mL), serum amyloid-A (SAA; mg/L), total antioxidant status (TAS; mmol/L), and malondialdehyde (MDA; μM) in non-reactor (single intradermal tuberculin test/SITT and IFN-γ release assay/IGRA negatives; *n* = 56) and reactor goats (SITT or IGRA positives; *n* = 34). Solid line represents the median and dashed lines represents the 25th (lower) and 75th (upper) percentiles.

Regarding APPs determination, no significant differences were observed in Hp (*p* > 0.05; MD = 0.04) and SAA (*p* > 0.05; MD = 0.3) levels between reactor and non-reactor goats (Table 3; Figure 3). In addition, no correlation (r_s_ = 0.18; *p* > 0.05) was observed between serum Hp and SAA levels. Moreover, the correlation between the baseline plasma levels of proinflammatory cytokines (IL-1β, IL-6 and TNF-α) and those of serum APPs (Hp and SAA) ranged from weak to null for all comparisons. Regarding the OS response, a significantly higher serum TAS (*p* = 0.014; MD = 0.1) was found in reactor goats compared to non-reactor, while no significant differences (*p* > 0.05; MD = −6.4) were observed for serum MDA (Table 3; Figure 3). Furthermore, no correlation (r_s_ = 0.10; *p* > 0.05) was observed between serum TAS and MDA.

**Table 3:** Median and interquartile range (IQR) of haptoglobin (Hp), serum amyloid A (SAA), total antioxidant status (TAS) and malondialdehyde (MDA) concentrations in reactor (single intradermal tuberculin test/SITT or IFN-γ release assay/IGRA positives; *n* = 34) and non-reactor goats (SITT and IGRA negatives; *n* = 56), median differences (MD) and statistical differences between them.

Marker	Reactor		Non-reactor		MD	Mann–Whitney U test
	Median	IQR	Median	IQR		(*p*-value)
Hp (mg/mL)	0.2	0.1–0.2	0.2	0.1–0.2	0.04	0.1
SAA (mg/L)	0.5	0.09–7.5	0.1	0.01–2.2	0.3	0.067
TAS (mmol/L)	1.2	1.1–1.3	1.1	0.9–1.2	0.1	**0.014**
MDA (uM)	15.9	0.0–43.8	22.3	0.00–52.7	−6.4	0.2

*p*-values <0.05 are marked in bold.

CBC results are shown in Table 4. No significant differences were found between non-reactor and reactor goats for any of the hematological parameters except for the percentage of mid-range cells (Mid%), which was significantly lower (*p* = 0.021; MD = −1.4) in reactor goats compared to non-reactors.

**Table 4:** Results of complete blood counts (CBC) in reactor (single intradermal tuberculin test/SITT or IFN-γ release assay/IGRA positives; *n* = 21) and non-reactor goats (SITT and IGRA negatives; *n* = 23) expressed as median and interquartile range (IQR), median differences (MD) and statistical differences between them.

Parameter	Reactor		Non-reactor		MD	Mann–Whitney U test
	Median	IQR	Median	IQR		(*p*-value)
WBC (×10^3^/ul)	16.4	13.7–18.1	16.0	13.2–18.6	0.3	0.8
Lym (×10^3^/ul l)	6.0	5.4–7.0	6.5	5.1–7.1	−0.5	0.9
Gran (×10^3^/ul)	8.2	6.4–10.0	8.3	6.3–9.8	−0.09	0.7
Mid (×10^3^/ul)	1.3	1.1–1.4	1.6	1.2–1.8	−0.2	0.1
Lym (%)	38.9	31.4–49.5	40.5	32.0–43.2	−1.6	0.6
Gran (%)	49.8	42.9–59.7	50.9	47.1–58.5	−1.1	0.8
Mid (%)	8.3	7.7–9.0	9.7	8.2–10.6	−1.4	**0.021**
RBC (×10^6^/ul)	10.3	9.9–11.1	10.6	10.0–11.5	−0.3	0.8
PCV (%)	20.9	18.5–22.2	21.9	18.5–22.4	−1.0	0.6
HGB (g/dL)	9.8	9.0–10.5	9.9	8.9–10.4	−0.1	0.8
MCV (fL)	19.4	18.8–21.0	19.3	18.1–20.4	0.1	0.4
MCH (pg)	9.1	8.9–9.7	9.3	9.0–9.5	−0.2	0.5
MCHC (g/dL)	48.3	41.7–51.2	46.9	44.2–52.0	1.4	0.3
RDW-CV	30.9	29.0–33.4	30.8	27.5–33.0	0.1	0.7
RDW-SD	21.9	18–22.7	19.8	17.8–23.1	2.1	0.3
PLT (×10^3^/ul)	51.0	28.0–74.0	75.0	34.5–148.5	−24.0	0.1
MPV (fL)	3.6	3.5–3.7	3.6	3.5–3.6	0.00	0.1
PDW	3.0	2.4–3.0	3.0	3.0–3.0	0.00	0.2
PCT (%)	0.02	0.01–0.02	0.02	0.01–0.05	0.00	0.1

*p*-values <0.05 are marked in bold.

## 4 Discussion

An in-depth characterization of the immune response against MTBC infection in goats is crucial for improving diagnosis of TB and thus optimize control strategies. The existing limitations of current techniques, such as the intradermal tests, can result in misdiagnosis of both infected and non-infected animals and contribute to TB persistence (Bezos et al., 2014b, 2012b; Gortazar et al., 2005; Alvarez et al., 2008). In this sense, the identification of specific biomarkers may enhance diagnostic accuracy, provide insights into host-pathogen interactions and contribute to the development of novel diagnostic tools.

In our study, levels of plasma IFN-γ, IL-1α, IL-6, IL-8, IL-10, MIP-1α, IL-36RA, IP-10 and VEGF-A in reactor goats varied significantly when whole blood samples were stimulated with PPDb compared to controls (PBS). However, in PPDb-stimulated samples only IFN-γ and IP-10 levels were significantly higher in reactor vs. non-reactor goats. These findings indicate a differential expression of IFN-γ and IP-10 in reactor goats to TB tests. The usefulness of IFN-γ-based assays has been well demonstrated in the past, and led to the implementation of the IGRA as an official technique aimed at maximizing the detection of TB infected animals in bTB eradication programs within the European Union (EFSA Panel on Animal Health and Welfare (AHAW), 2012; Wood and Jones, 2001; Liébana et al., 1998). In goats, IGRAs are also a more sensitive technique than the intradermal tests (Roy et al., 2020), although its use for routine diagnosis of cTB has not been implemented yet (Ministerio de Agricultura, Pesca y Alimentación, 2024). In our study, a strong correlation was observed between IGRA results (OD) and the levels of IFN-γ in plasma samples stimulated with PPDb. In contrast, in a study from Ortega et al. (2022), a weak correlation was observed between OD values of IFN-γ in PPDb-stimulated plasma and the serum levels of this cytokine in samples collected 3 days after an intradermal injection of PPDb. Despite PPDb intradermal inoculation could boost IFN-γ responses, this effect has been mainly described under experimental infection settings (Jones et al., 2017; Schiller et al., 2010) and discrepancies between studies may be due to the effect of *in vitro* lymphocyte stimulation by PPDb.

Our results also suggest IP-10 could have potential as a biomarker of infection in goats. IP-10, a chemokine strongly induced by IFN-γ, is crucial in delayed-type hypersensitivity responses to mycobacterial antigens (Kaplan et al., 1987). IP-10-based assays seems to perform similar to IFN-γ-based in cattle (Coad et al., 2019). In goats, Cooke et al. (2023) observed that the performance of an in-house bovine IP-10-based ELISA using plasma stimulated with mycobacterial specific antigens (ESAT-6 and CFP-10) in goats was very similar to IGRA in terms of sensitivity and specificity, though no information was available regarding the IP-10 production in PPDb-stimulated plasma. In our study, PPDb-IP-10 and Δ-IP-10 levels were significantly higher in reactor goats compared to non-reactors, which may indicate a relationship between IP-10 and the reactivity to TB diagnostic techniques, probably due to an infection-dependent release. Furthermore, a very strong correlation was found in our study between the levels of IP-10 and IFN-γ in plasma samples after PPDb stimulation or when considering the difference PPDb-PBS, as previously described in cattle (Parsons et al., 2016; Li et al., 2022). Recent studies in TB infected cattle (Parsons et al., 2016; Li et al., 2022; Palmer et al., 2020) and African buffalo (Smith et al., 2021a; Goosen et al., 2015) have shown high levels of IP-10 expression after PPDb stimulation of whole blood samples, sometimes also exceeding those levels of IFN-γ. In our study the IP-10 levels in plasma samples after PPDb stimulation exceeded those of IFN-γ. This could be related to the high baseline levels of IP-10 that may be spontaneously induced by interferons and proinflammatory cytokines (Palmer et al., 2020; Parsons et al., 2016; Waters et al., 2012). Therefore, measurement of Δ-IP-10 would be a preferred indicator of infection and, in fact, these levels in reactor goats were higher than in non-reactor for all cases in our study. However, a limitation of the present study is the absence of post-mortem results to enable more definitive interpretation of the results, though the sensitivity of these tests are not perfect as well (Gomez-Buendia et al., 2023a; Jiménez-Martín et al., 2025).

Moreover, although IL-1α plasma levels in samples stimulated with PPDb in reactor goats did not significantly exceed those found in non-reactors, significant differences were observed in the level of Δ-IL-1α. IL-1α is a pro-inflammatory cytokine released at the early stages of infection aimed at enhancing the release of several chemokines and inflammation process (Mantovani et al., 2019). Our results are consistent with a study from Franzoni et al. (2024), in which higher levels of Δ-IL-1α were detected in IGRA-reactor water buffalo compared to non-reactor. In addition, Smith et al. (2021a), observed no differences in the level of IL-1α in plasma stimulated with ESAT-6/CFP-10 compared to non-stimulated in African buffalo infected with *M. bovis*, though differences with non-infected buffalo could not be evaluated since an uninfected group was not included in the study. Although no differences in the levels of IL-1α between reactor vs. non-reactor goats were observed in PBS or PPDb-stimulated plasma, the differences found between these groups in Δ-IL-1α levels suggest its potential as biomarker of cTB infection. Furthermore, other studies in cattle and buffalo have found a significant increase in the expression of IL-1β (Palmer et al., 2020; Elnaggar et al., 2017; Jones et al., 2010), IL-4 (Thacker et al., 2007), IL-8 (Blanco et al., 2009), IL-10 (Widdison et al., 2006) or IL-17A (Smith et al., 2021a; Franzoni et al., 2024; Mensikova et al., 2013) in bTB infected animals compared to uninfected. In contrast, no differences were found here regardless of the reactivity status to TB techniques. Variability among studies may be due to a different role of these cytokines in cTB infected animals (Palmer et al., 2020) or to relatively small sample size here, although it was comparable to previous studies (Palmer et al., 2020; Smith et al., 2021a; Franzoni et al., 2024; Steinbach et al., 2021). In this sense, despite we observed almost significant differences in the level of Δ-VEGF-A in reactor vs. non-reactor goats, more work needs to be done to understand the role of this cytokine in cTB.

APPs are blood proteins produced in animals subjected to internal or external challenges (Saco and Bassols, 2023; Eckersall and Bell, 2010) and provide valuable diagnostic information as regards diagnosis and monitoring of different infectious diseases (Murata et al., 2004; Petersen et al., 2004; El-Deeb et al., 2019), including TB (Kumar et al., 2021). In this context, increased serum Hp levels and systemic AA-amyloidosis (resulting from SAA deposits) associated with TB have been reported in wildlife species (Vicente et al., 2019; Segalés et al., 2005). In our study, the reactivity to TB diagnostic tests do not appear to be associated with Hp and SAA levels since no differences were found between reactor and non-reactor goats. However, it is known that APPs are highly sensitive, non-specific indicator of inflammation (Eckersall, 2008; Didkowska et al., 2025). In this sense, goats from our study may be affected by other metabolic or infectious diseases, precluding differences in APPs levels between groups been observed. Therefore, usefulness of APPs for diagnosis of cTB would be limited.

OS is represents a key defense mechanisms of macrophages against mycobacterial multiplication, although it may also cause host-tissue damage as a side effect (Shastri et al., 2018; Verma et al., 2014). In experimental conditions, infection of guinea pigs with *Mycobacterium tuberculosis* resulted in a decrease in serum antioxidant capacity (TAS) due to depletion of antioxidant agents and an increase in lipid peroxidation (MDA) (Palanisamy et al., 2011). In contrast, our study revealed significantly higher levels of serum TAS in reactor goats compared to non-reactor, whereas no differences in the MDA levels were found. This increase in TAS may reflect a compensatory antioxidant response, as reported in other infectious diseases (Yehia et al., 2024; Baptistiolli et al., 2018). However, TAS and MDA may be influenced by several internal and external factors (Gassó et al., 2016; Blanca et al., 2024; Pellegrini et al., 2020). Gassó et al. (2016) observed that levels of antioxidant agents and MDA in free-ranging wild boar were primarily influenced by host-related and environmental factors rather than by TB infection status itself. Thus, TAS and MDA cannot be considered as unique biomarkers of individual antioxidant status and seem not be specifically related with the reactivity to TB diagnostic techniques in goats.

Finally, hematological parameters (CBC) were also evaluated in our study and no significant differences related to the infection status based on diagnostic reactivity were observed except for the percentage of mid-range cells (Mid%). Nevertheless, since differences in the total count of mid-range cells or in other white-cell line were not found, these results should be carefully interpreted. In line with our findings, Bezos et al. (2012a) did not found significant differences in white-cells counts between goats infected with *M. caprae* and non-infected controls. Similarly, Miller et al. (1989) observed no differences in CBC parameters between *M. bovis* infected bison and healthy controls, and also Thapa et al. (2021) did not found differences in hematological parameters between reactor and non-reactor elephants to a TB humoral test. Therefore, CBC does not seem to be a good predictor of TB infection status.

In conclusion, we demonstrated that in addition to IFN-γ, IP-10 and IL-1α are also associated with the reactivity to TB tests and suggest their potential as biomarkers of cTB infection. While the measurement of TAS may provide information about the general health status of the goats, its usefulness in diagnostic terms would be more limited.

## Data Availability

The original contributions presented in the study are included in the article/Supplementary material, further inquiries can be directed to the corresponding author.

## References

[ref1] Aguilar Diaz De Leon J. Borges C. R. (2020). Evaluation of oxidative stress in biological samples using the thiobarbituric acid reactive substances assay. J. Vis. Exp. 12:10.3791/61122. doi: 10.3791/61122, 32478759 PMC9617585

[ref2] Agulló-Ros I. Vaz-Rodrigues R. Domínguez M. Roy Á. Ortega J. Moreno I. . (2025). Immunological mechanisms involved in the protection against development of pulmonary tuberculosis in naturally infected goats. Vet. Microbiol. 300:110320. doi: 10.1016/j.vetmic.2024.110320, 39626442

[ref3] Al-Hassan M. J. (2018). Antioxidant biomarkers in the milk of early postpartum Aardi goats during winter. Small Rumin. Res. 162, 8–11. doi: 10.1016/j.smallrumres.2018.03.011

[ref4] Alvarez J. de Juan L. Bezos J. Romero B. Sáez J. L. Reviriego Gordejo F. J. . (2008). Interference of paratuberculosis with the diagnosis of tuberculosis in a goat flock with a natural mixed infection. Vet. Microbiol. 128, 72–80. doi: 10.1016/j.vetmic.2007.08.034 17954015

[ref5] Aranaz A. Cousins D. Mateos A. Domínguez L. (2003). Elevation of *Mycobacterium tuberculosis* subsp. *caprae* Aranaz et al. 1999 to species rank as *Mycobacterium caprae* comb. nov., sp. nov. Int. J. Syst. Evol. Microbiol. 53, 1785–1789. doi: 10.1099/ijs.0.02532-0 14657105

[ref6] Balepur S. S. Schlossberg D. (2016). Hematologic complications of tuberculosis. Microbiol. Spectr. 4:10-1128. doi: 10.1128/microbiolspec.TNMI7-0004-2016 28084210

[ref7] Baptistiolli L. Narciso L. G. Almeida B. F. M. Bosco A. M. Souza J. C. Torrecilha R. B. P. . (2018). Systemic oxidative stress in Suffolk and Santa Ines sheep experimentally infected with *Haemonchus contortus*. Acta Parasitol. 63, 504–514. doi: 10.1515/ap-2018-0060, 29975652

[ref8] Beghelli D. Lupidi G. Damiano S. Cavallucci C. Bistoni O. De Cosmo A. (2016). Rapid assay to evaluate the total antioxidant capacity in donkey milk and in more common animal milk for human consumption. Austin Food Sci. 1:1003.

[ref9] Bernitz N. Kerr T. J. Goosen W. J. Clarke C. Higgitt R. Roos E. O. . (2019). Parallel measurement of IFN-γ and IP-10 in QuantiFERON^®^-TB gold (QFT) plasma improves the detection of *Mycobacterium bovis* infection in African buffaloes (*Syncerus caffer*). Prev. Vet. Med. 169:104700. doi: 10.1016/j.prevetmed.2019.104700, 31311648

[ref10] Bezos J. Álvarez J. Moreno I. de Juan L. Romero B. Rodríguez S. . (2012a). Study of peripheral blood cell populations involved in the immune response of goats naturally infected with *Mycobacterium caprae*. Res. Vet. Sci. 93, 163–167. doi: 10.1016/j.rvsc.2011.05.020, 21689835

[ref11] Bezos J. Alvarez J. Romero B. Aranaz A. Juan L. (2012b). Tuberculosis in goats: assessment of current in vivo cell-mediated and antibody-based diagnostic assays. Vet. J. 191, 161–165. doi: 10.1016/j.tvjl.2011.02.010, 21388843

[ref12] Bezos J. Casal C. Romero B. Schroeder B. Hardegger R. Raeber A. J. . (2014a). Current ante-mortem techniques for diagnosis of bovine tuberculosis. Res. Vet. Sci. 97, S44–S52. doi: 10.1016/j.rvsc.2014.04.002 24768355

[ref13] Bezos J. Marqués S. Álvarez J. Casal C. Romero B. Grau A. . (2014b). Evaluation of single and comparative intradermal tuberculin tests for tuberculosis eradication in caprine flocks in Castilla y León (Spain). Res. Vet. Sci. 96, 39–46. doi: 10.1016/j.rvsc.2013.10.007, 24239314

[ref14] Blanca P. M. María Luisa F. R. Guadalupe M. Fátima C. L. (2024). Oxidative stress in canine diseases: a comprehensive review. Antioxidants 13:1396. doi: 10.3390/antiox13111396, 39594538 PMC11591346

[ref15] Blanco F. C. Schierloh P. Bianco M. V. Caimi K. Meikle V. Alito A. E. . (2009). Study of the immunological profile towards *Mycobacterium bovis* antigens in naturally infected cattle. Microbiol. Immunol. 53, 460–467. doi: 10.1111/j.1348-0421.2009.00141.x, 19659930

[ref16] Coad M. Doyle M. Steinbach S. Gormley E. Vordermeier M. Jones G. (2019). Simultaneous measurement of antigen-induced CXCL10 and IFN-γ enhances test sensitivity for bovine TB detection in cattle. Vet. Microbiol. 230, 1–6. doi: 10.1016/j.vetmic.2019.01.007, 30827373

[ref17] Cooke D. M. Goosen W. J. Burgess T. Witte C. Miller M. A. (2023). *Mycobacterium tuberculosis* complex detection in rural goat herds in South Africa using Bayesian latent class analysis. Vet. Immunol. Immunopathol. 257:110559. doi: 10.1016/j.vetimm.2023.110559, 36739737

[ref18] De La Rua-Domenech R. Goodchild A. T. Vordermeier H. M. Hewinson R. G. Christiansen K. H. Clifton-Hadley R. S. (2006). Ante mortem diagnosis of tuberculosis in cattle: a review of the tuberculin tests, γ-interferon assay and other ancillary diagnostic techniques. Res. Vet. Sci. 81, 190–210. doi: 10.1016/j.rvsc.2005.11.005, 16513150

[ref19] de Val P. B. López-Soria S. Nofrarías M. Martín M. Vordermeier H. M. Villarreal-Ramos B. . (2011). Experimental model of tuberculosis in the domestic goat after endobronchial infection with *Mycobacterium caprae*. Clin. Vaccine Immunol. 18, 1872–1881. doi: 10.1128/CVI.05323-11, 21880849 PMC3209027

[ref20] Didkowska A. Klich D. Matusik K. Wilczek M. Anusz K. Krajewska-Wędzina M. (2025). Haptoglobin response in serum and pleural fluid of tuberculin reactor cattle assessed by culture and gross pathology. Vet. J. 314:106453. doi: 10.1016/j.tvjl.2025.106453, 41015378

[ref21] Domingo M. Vidal E. Marco A. (2014). Pathology of bovine tuberculosis. Res. Vet. Sci. 97, S20–S29. doi: 10.1016/j.rvsc.2014.03.017 24731532

[ref22] Eckersall P. D. (2004). The time is right for acute phase protein assays. Vet. J. 168, 3–5. doi: 10.1016/j.tvjl.2003.09.003 15158201

[ref23] Eckersall P. D. (2008). “Proteins, proteomics, and the dysproteinemias” in Clinical biochemistry of domestic animals. eds. Kaneko J. J. . (Amsterdam, Netherlands: Elsevier), 117–155.

[ref24] Eckersall P. D. Bell R. (2010). Acute phase proteins: biomarkers of infection and inflammation in veterinary medicine. Vet. J. 185, 23–27. doi: 10.1016/j.tvjl.2010.04.009 20621712

[ref25] EFSA Panel on Animal Health and Welfare (AHAW) (2012). Scientific opinion on the use of a gamma interferon test for the diagnosis of bovine tuberculosis. EFSA J. 10:2975. doi: 10.2903/j.efsa.2012.2975

[ref26] El-Deeb W. Ghoneim I. Fayez M. Elsohaby I. Alhaider A. ElGioushy M. (2019). Acute phase proteins, proinflammatory cytokines and oxidative stress biomarkers in sheep, goats and she-camels with *Coxiella burnetii* infection-induced abortion. Comp. Immunol. Microbiol. Infect. Dis. 67:101352. doi: 10.1016/j.cimid.2019.101352, 31568899

[ref27] Elnaggar M. M. Abdellrazeq G. S. Elsisy A. Mahmoud A. H. Shyboub A. Sester M. . (2017). Evaluation of antigen specific interleukin-1β as a biomarker to detect cattle infected with *Mycobacterium bovis*. Tuberculosis 105, 53–59. doi: 10.1016/j.tube.2017.04.009, 28610788

[ref28] Erel O. (2004). A novel automated direct measurement method for total antioxidant capacity using a new generation, more stable ABTS radical cation. Clin. Biochem. 37, 277–285. doi: 10.1016/j.clinbiochem.2003.11.015, 15003729

[ref29] Etna M. P. Giacomini E. Severa M. Coccia E. M. (2014). Pro- and anti-inflammatory cytokines in tuberculosis: a two-edged sword in TB pathogenesis. Semin. Immunol. 26, 543–551. doi: 10.1016/j.smim.2014.09.011, 25453229

[ref30] Eurostat. (2025). Goats population – annual data. Available online at: https://ec.europa.eu/eurostat/databrowser/view/APRO_MT_LSGOAT/default/table?lang=en (accessed September 16, 2025).

[ref31] Franzoni G. Signorelli F. Mazzone P. Donniacuo A. De Matteis G. Grandoni F. . (2024). Cytokines as potential biomarkers for the diagnosis of *Mycobacterium bovis* infection in Mediterranean buffaloes (*Bubalus bubalis*). Front. Vet. Sci. 11:1512571. doi: 10.3389/fvets.2024.1512571, 39776597 PMC11703857

[ref32] Gassó D. Vicente J. Mentaberre G. Soriguer R. Jiménez Rodríguez R. Navarro-González N. . (2016). Oxidative stress in wild boars naturally and experimentally infected with *Mycobacterium bovis*. PLoS One 11:e0163971. doi: 10.1371/journal.pone.0163971, 27682987 PMC5040450

[ref33] Gomez-Buendia A. Pozo P. Picasso-Risso C. Branscum A. Perez A. Alvarez J. (2023a). Accuracy of tests for diagnosis of animal tuberculosis: moving away from the golden calf (and towards Bayesian models). Transbound. Emerg. Dis. 2023:7615716. doi: 10.1155/2023/7615716, 40303756 PMC12017179

[ref34] Gomez-Buendia A. Romero B. Bezos J. Saez J. L. Archetti I. Pacciarini M. L. . (2023b). Evaluation of the performance of the IFN-γ release assay in bovine tuberculosis free herds from five European countries. Vet. Res. 54:55. doi: 10.1186/s13567-023-01187-5, 37403088 PMC10320920

[ref35] González F. H. Tecles F. Martínez-Subiela S. Tvarijonaviciute A. Soler L. Cerón J. J. (2008). Acute phase protein response in goats. J. Vet. Diagn. Invest. 20, 580–584. doi: 10.1177/104063870802000507, 18776089

[ref36] Goosen W. J. Cooper D. Miller M. A. van Helden P. D. Parsons S. D. (2015). IP-10 is a sensitive biomarker of antigen recognition in whole-blood stimulation assays used for the diagnosis of *Mycobacterium bovis* infection in African buffaloes (*Syncerus caffer*). Clin. Vaccine Immunol. 22, 974–978. doi: 10.1128/CVI.00324-15, 26108287 PMC4519731

[ref37] Gortazar C. Vicente J. Samper S. Garrido J. M. Fernández-De-Mera I. G. Gavín P. . (2005). Molecular characterization of *Mycobacterium tuberculosis* complex isolates from wild ungulates in south-Central Spain. Vet. Res. 36, 43–52. doi: 10.1051/vetres:2004051, 15610722

[ref38] Hari-Dass R. Shah C. Meyer D. J. Raynes J. G. (2005). Serum amyloid a protein binds to outer membrane protein a of gram-negative bacteria. J. Biol. Chem. 280, 18562–18567. doi: 10.1074/jbc.M500490200 15705572

[ref39] Iliev P. Georgieva T. (2018). Acute phase proteins in sheep and goats – function, reference ranges and assessment methods: an overview. Bulg. J. Vet. Med. 21, 1–16. doi: 10.15547/bjvm.1050

[ref40] Jiménez-Martín D. Muñoz-Fernández L. Agulló-Ros I. Jiménez-Pizarro N. Cano-Terriza D. García-Bocanegra I. . (2025). Evaluation of techniques for post-mortem diagnosis of *Mycobacterium tuberculosis* complex infection in goats. Vet. Microbiol. 304:110485. doi: 10.1016/j.vetmic.2025.110485, 40138988

[ref41] Jones G. J. Coad M. Khatri B. Bezos J. Parlane N. A. Buddle B. M. . (2017). Tuberculin skin testing boosts interferon gamma responses to DIVA reagents in *Mycobacterium bovis*-infected cattle. Clin. Vaccine Immunol. 24:e00551-16. doi: 10.1128/CVI.00551-16, 28331078 PMC5424240

[ref42] Jones G. J. Pirson C. Hewinson R. G. Vordermeier H. M. (2010). Simultaneous measurement of antigen-stimulated interleukin-1 beta and gamma interferon production enhances test sensitivity for the detection of *Mycobacterium bovis* infection in cattle. Clin. Vaccine Immunol. 17, 1946–1951. doi: 10.1128/CVI.00377-10, 20926697 PMC3008179

[ref43] Kaplan G. Luster A. D. Hancock G. Cohn Z. A. (1987). The expression of a gamma interferon-induced protein (IP-10) in delayed immune responses in human skin. J. Exp. Med. 166, 1098–1108. doi: 10.1084/jem.166.4.1098, 2443597 PMC2188712

[ref44] Kumar N. P. Moideen K. Nancy A. Viswanathan V. Thiruvengadam K. Sivakumar S. . (2021). Acute phase proteins are baseline predictors of tuberculosis treatment failure. Front. Immunol. 12:731878. doi: 10.3389/fimmu.2021.731878, 34867953 PMC8634481

[ref45] Li X. Xia A. Xu Z. Liu J. Fu S. Cao Z. . (2022). Development and evaluation of a *Mycobacterium bovis* interferon-γ enzyme-linked immunospot (ELISpot) assay for detection of bovine tuberculosis. J. Dairy Sci. 105, 6021–6029. doi: 10.3168/jds.2021-21301, 35570041

[ref46] Liébana E. Aranaz A. Urquía J. J. Mateos A. Domínguez L. (1998). Evaluation of the gamma-interferon assay for eradication of tuberculosis in a goat herd. Aust. Vet. J. 76, 50–53. doi: 10.1111/j.1751-0813.1998.tb15686.x, 9578768

[ref47] Lowie T. Jourquin S. Stuyvaert S. Hanley-Cook G. Pardon B. (2025). Associations between lung consolidation and serum amyloid a and haptoglobin, and the potential of acute phase proteins to differentiate primary respiratory tract pathogens in calves. J. Dairy Sci. 108, 943–953. doi: 10.3168/jds.2024-25093, 39414004

[ref48] Malone K. M. Gordon S. V. (2017). *Mycobacterium tuberculosis* complex members adapted to wild and domestic animals. Adv. Exp. Med. Biol. 1019, 135–154. doi: 10.1007/978-3-319-64371-7_7, 29116633

[ref49] Mantovani A. Dinarello C. A. Molgora M. Garlanda C. (2019). Interleukin-1 and related cytokines in the regulation of inflammation and immunity. Immunity 50, 778–795. doi: 10.1016/j.immuni.2019.03.012, 30995499 PMC7174020

[ref50] Martínez-Lirola M. Herranz M. Buenestado Serrano S. Rodríguez-Grande C. Dominguez Inarra E. Garrido-Cárdenas J. A. . (2023). A one health approach revealed the long-term role of *Mycobacterium caprae* as the hidden cause of human tuberculosis in a region of Spain, 2003 to 2022. Euro Surveill. 28:2200852. doi: 10.2807/1560-7917.ES.2023.28.12.2200852, 36951787 PMC10037661

[ref51] Mensikova M. Stepanova H. Faldyna M. (2013). Interleukin-17 in veterinary animal species and its role in various diseases: a review. Cytokine 64, 11–17. doi: 10.1016/j.cyto.2013.06.002, 23810220

[ref52] Miller L. D. Thoen C. O. Throlson K. J. Himes E. M. Morgan R. L. (1989). Serum biochemical and hematologic values of normal and *Mycobacterium bovis*-infected American bison. J. Vet. Diagn. Invest. 1, 219–222. doi: 10.1177/104063878900100304, 2488347

[ref53] Ministerio de Agricultura, Pesca y Alimentación. Manual para el control de la infección por el CMT en establecimientos de ganado caprino incluidos en el programa nacional de erradicación de la infección por el complejo *Mycobacterium tuberculosis* (CMT). (2024). Available online at: https://www.mapa.gob.es/en/ganaderia/temas/sanidad-animal-higiene-ganadera/8manualcaprino2024_tcm38-553693.pdf (accessed September 16, 2025)

[ref54] Murata H. Shimada N. Yoshioka M. (2004). Current research on acute phase proteins in veterinary diagnosis: an overview. Vet. J. 168, 28–40. doi: 10.1016/S1090-0233(03)00119-9, 15158206

[ref55] Ortega J. de Juan L. Sevilla I. A. Garrido J. M. Roy Á. Velasco C. . (2022). Effect of a recent parenteral dexamethasone and ketoprofen administration on the immunological diagnosis of tuberculosis in goats. Front. Vet. Sci. 9:1042428. doi: 10.3389/fvets.2022.1042428, 36439353 PMC9686350

[ref56] Palanisamy G. S. Kirk N. M. Ackart D. F. Shanley C. A. Orme I. M. Basaraba R. J. (2011). Evidence for oxidative stress and defective antioxidant response in guinea pigs with tuberculosis. PLoS One 6:e26254. doi: 10.1371/journal.pone.002625431 22028843 PMC3196542

[ref57] Palmer M. V. Kanipe C. Boggiatto P. M. (2022). The bovine tuberculoid granuloma. Pathogens 11:61. doi: 10.3390/pathogens11010061, 35056009 PMC8780557

[ref58] Palmer M. V. Thacker T. C. Rabideau M. M. Jones G. J. Kanipe C. Vordermeier H. M. . (2020). Biomarkers of cell-mediated immunity to bovine tuberculosis. Vet. Immunol. Immunopathol. 220:109988. doi: 10.1016/j.vetimm.2019.109988, 31846797

[ref59] Parsons S. D. McGill K. Doyle M. B. Goosen W. J. van Helden P. D. Gormley E. (2016). Antigen-specific IP-10 release is a sensitive biomarker of *Mycobacterium bovis* infection in cattle. PLoS One 11:e0155440. doi: 10.1371/journal.pone.0155440, 27167122 PMC4864312

[ref60] Pellegrini N. Vitaglione P. Granato D. Fogliano V. (2020). Twenty-five years of total antioxidant capacity measurement of foods and biological fluids: merits and limitations. J. Sci. Food Agric. 100, 5064–5078. doi: 10.1002/jsfa.9550 30578632

[ref61] Pérez De Val B. Vidal E. Stuber T. Sáez J. L. Tórtola M. T. (2025). Zoonotic tuberculosis in Catalonia, Spain: phylogenetic insights into *Mycobacterium bovis* and *M. caprae* transmission at the human-livestock interface. One Health 20:100993. doi: 10.1016/j.onehlt.2025.100993, 40035021 PMC11875796

[ref62] Perez-Montero B. Fermin-Rodriguez M. L. Portero-Fuentes M. Sarquis J. Caceres S. Del Portal J. C. I. . (2024). Serum total antioxidant status in dogs: reference intervals and influence of multiple biological and analytical factors. Vet. Clin. Pathol. 53, 399–408. doi: 10.1111/vcp.13381, 39396933 PMC11735657

[ref63] Perez-Montero B. Fermin-Rodriguez M. L. Portero-Fuentes M. Sarquis J. Caceres S. Portal J. C. I. D. . (2025). Malondialdehyde (MDA) and 8-hydroxy-2′-deoxyguanosine (8-OHdG) levels in canine serum: establishing reference intervals and influencing factors. BMC Vet. Res. 21:161. doi: 10.1186/s12917-025-04614-1, 40069799 PMC11900598

[ref64] Petersen H. H. Nielsen J. P. Heegaard P. M. (2004). Application of acute phase protein measurements in veterinary clinical chemistry. Vet. Res. 35, 163–187. doi: 10.1051/vetres:2004002, 15099494

[ref65] R Core Team. R. A language and environment for statistical computing. (2025). Available online at: https://www.R-project.org (accessed September 16, 2025).

[ref66] Roy A. Infantes-Lorenzo J. A. De La Cruz M. L. Domínguez L. Álvarez J. Bezos J. (2020). Accuracy of tuberculosis diagnostic tests in small ruminants: a systematic review and meta-analysis. Prev. Vet. Med. 182:105102. doi: 10.1016/j.prevetmed.2020.105102, 32739695

[ref67] Saco Y. Bassols A. (2023). Acute phase proteins in cattle and swine: a review. Vet. Clin. Pathol. 52, 50–63. doi: 10.1111/vcp.13220 36526287

[ref68] Schiller I. Vordermeier H. M. Waters W. R. Whelan A. O. Coad M. Gormley E. . (2010). Bovine tuberculosis: effect of the tuberculin skin test on in vitro interferon gamma responses. Vet. Immunol. Immunopathol. 136, 1–11. doi: 10.1016/j.vetimm.2010.02.007, 20219253

[ref69] Schiller I. Waters W. R. Vordermeier H. M. Nonnecke B. Welsh M. Keck N. . (2009). Optimization of a whole-blood gamma interferon assay for detection of *Mycobacterium bovis*-infected cattle. Clin. Vaccine Immunol. 16, 1196–1202. doi: 10.1128/CVI.00150-09, 19571108 PMC2725547

[ref70] Segalés J. Vicente J. Luján L. Toussaint M. J. Gruys E. Gortázar C. (2005). Systemic AA-amyloidosis in a European wild boar (*Sus scrofa*) suffering from generalized tuberculosis. J. Vet. Med. A Physiol. Pathol. Clin. Med. 52, 135–137. doi: 10.1111/j.1439-0442.2005.00703.x, 15836445

[ref71] Seva J. Hernández D. Bernabé A. Pallarés F. J. Navarro J. A. (2000). Immunophenotypical characterization of the lymphocyte infiltrate in caprine pulmonary tuberculosis. J. Comp. Pathol. 123, 96–103. doi: 10.1053/jcpa.2000.0397 11032661

[ref72] Shah A. R. Desai K. N. Maru A. M. (2022). Evaluation of hematological parameters in pulmonary tuberculosis patients. J. Family Med. Prim. Care 11, 4424–4428. doi: 10.4103/jfmpc.jfmpc_2451_21, 36353004 PMC9638606

[ref73] Shastri M. D. Shukla S. D. Chong W. C. Dua K. Peterson G. M. Patel R. P. . (2018). Role of oxidative stress in the pathology and management of human tuberculosis. Oxidative Med. Cell. Longev. 2018:7695364. doi: 10.1155/2018/7695364, 30405878 PMC6201333

[ref74] Smith K. Kleynhans L. Snyders C. Bernitz N. Cooper D. van Helden P. . (2021a). Use of the MILLIPLEX^®^ bovine cytokine/chemokine multiplex assay to identify *Mycobacterium bovis*-infection biomarkers in African buffaloes (*Syncerus caffer*). Vet. Immunol. Immunopathol. 231:110152. doi: 10.1016/j.vetimm.2020.110152, 33227620

[ref75] Smith K. Kleynhans L. Warren R. M. Goosen W. J. Miller M. A. (2021b). Cell-mediated immunological biomarkers and their diagnostic application in livestock and wildlife infected with *Mycobacterium bovis*. Front. Immunol. 12:639605. doi: 10.3389/fimmu.2021.639605, 33746980 PMC7969648

[ref76] Steinbach S. Jalili-Firoozinezhad S. Srinivasan S. Melo M. B. Middleton S. Konold T. . (2021). Temporal dynamics of intradermal cytokine response to tuberculin in *Mycobacterium bovis* BCG-vaccinated cattle using sampling microneedles. Sci. Rep. 11:7074. doi: 10.1038/s41598-021-86398-6, 33782422 PMC8007627

[ref77] Thacker T. C. Palmer M. V. Waters W. R. (2007). Associations between cytokine gene expression and pathology in *Mycobacterium bovis* infected cattle. Vet. Immunol. Immunopathol. 119, 204–213. doi: 10.1016/j.vetimm.2007.05.009, 17628695

[ref78] Thapa J. Mikota S. K. Gairhe K. P. Paudel S. Singh D. K. Dhakal I. P. . (2021). Tuberculosis seroprevalence and comparison of hematology and biochemistry parameters between seropositive and seronegative captive Asian elephants of Nepal. J. Vet. Med. Sci. 83, 1278–1283. doi: 10.1292/jvms.21-0113, 34108338 PMC8437712

[ref79] Turk R. Juretić D. Geres D. Svetina A. Turk N. Flegar-Mestrić Z. (2008). Influence of oxidative stress and metabolic adaptation on PON1 activity and MDA level in transition dairy cows. Anim. Reprod. Sci. 108, 98–106. doi: 10.1016/j.anireprosci.2007.07.012, 17850995

[ref80] Verma I. Surinder K. J. Nirmal K. G. (2014). “Oxidative stress in tuberculosis” in Studies on respiratory diseases. eds. Ganguly N. K. . (New York, NY: Springer), 101–114.

[ref81] Vicente J. Martinez-Guijosa J. Tvarijonaviciute A. Fernandez-de Mera I. G. Gortazar C. Ceron J. J. . (2019). Serum haptoglobin response in red deer naturally infected with tuberculosis. Comp. Immunol. Microbiol. Infect. Dis. 64, 25–30. doi: 10.1016/j.cimid.2019.01.021, 31174696

[ref82] Waters W. R. Thacker T. C. Nonnecke B. J. Palmer M. V. Schiller I. Oesch B. . (2012). Evaluation of gamma interferon (IFN-γ)-induced protein 10 responses for detection of cattle infected with *Mycobacterium bovis*: comparisons to IFN-γ responses. Clin. Vaccine Immunol. 19, 346–351. doi: 10.1128/CVI.05657-11, 22237891 PMC3294610

[ref83] Widdison S. Schreuder L. J. Villarreal-Ramos B. Howard C. J. Watson M. Coffey T. J. (2006). Cytokine expression profiles of bovine lymph nodes: effects of *Mycobacterium bovis* infection and bacille Calmette-Guérin vaccination. Clin. Exp. Immunol. 144, 281–289. doi: 10.1111/j.1365-2249.2006.03053.x, 16634802 PMC1809664

[ref84] Wood P. R. Jones S. L. (2001). <article-title update="added">BOVIGAMTM: an in vitro cellular diagnostic test for bovine tuberculosis. Tuberculosis 81, 147–155. doi: 10.1054/tube.2000.0272, 11463236

[ref85] Yehia S. G. Saad M. F. Mosallam T. E. Abdel-Mobdy A. E. Megahed E. A. Aly H. H. . (2024). Evaluation of oxidative stress, compositional and biochemical changes in milk and serum of cows with subclinical mastitis. Comp. Clin. Pathol. 33, 643–652. doi: 10.21203/rs.3.rs-1912881/v1

