# Supplementary material for: Exploring specific biomarkers in blood for in vitro diagnosis of caprine tuberculosis
Source: Front Microbiol. 2026 Feb 20;17:1765857. doi: 10.3389/fmicb.2026.1765857 (PMC12963342; doi:10.3389/fmicb.2026.1765857)
Supplement: Supplementary file 1 [file Table_1.docx]

Supplementary Material

# Supplementary Tables

**Table S1.** Median [and interquartile range] of plasma cytokine concentrations (pg/mL) measured in reactor (single intradermal tuberculin test/SITT or IFN-γ Release Assay/IGRA positives; n=10) and non-reactor goats (SITT and IGRA negatives; n=9).

| Cytokine^a^ | Reactor | |  |  | Non-reactor | |  |
| --- | --- | --- | --- | --- | --- | --- | --- |
|  | PBS | PPDb | Δ-cytokine^b^ |  | PBS | PPDb | Δ-cytokine^b^ |
| IFN-γ | 0.2 [0.0-1.1] | 45.1 [7.14-105.9] | 44.0 [7.1-105.5] |  | 0.0 [0.0-0.0] | 0.0 [0.0-0.0] | 0.0 [0.0-0.0] |
| IL-1α | 26.7 [12.6-40.3] | 58.52 [27.1-104.6] | 21.1 [13.6-81.6] |  | 21.8 [5.7-50.8] | 30.3 [12.0-37.1] | 5.3 [(-)0.4-15.3] |
| IL-1β | 0.0 [0.0-0.0] | 0.0 [0.0-0.0] | 0.0 [0.0-0.0] |  | 0.0 [0.0-0.0] | 0.0 [0.0-0.0] | 0.0 [0.0-0.0] |
| IL-4 | 158.1 [107.2-199.0] | 156.0 [94.3-205.2] | (-)1.2 [(-)10.3-12.8] |  | 159.5 [130.7-258.6] | 162.9 [124.2-248.4] | 3.3 [(-)6.5-11.0] |
| IL-6 | 93.1 [54.8-158.9] | 553.5 [444.4-1,115.9] | 500.5 [360.8-951.6] |  | 98.4 [57.8-236.9] | 498.0 [427.2-536.0] | 393.2 [(-)32.05-467.06] |
| IL-8 | 1,641.6 [1,496.3-2,096.3] | 2,139.2 [1,723.0-2,776.0] | 311.3 [162.0-572.1] |  | 1,826.8 [1,420.5-2,338.8] | 1,915.5 [1,665.9-2,266.4] | 121.5 [0.0-495.0] |
| IL-10 | 82.7 [59.3-110.0] | 130.2 [96.9-207.4] | 43.8 [19.7-61.8] |  | 59.2 [47.4-99.8] | 97.0 [75.1-102.7] | 27.4 [19.5-37.3] |
| IL-17A | 0.0 [0.0-0.2] | 0.3 [0.0-0.6] | 0.1 [0.0-0.4] |  | 0.3 [0.0-0.4] | 0.4 [0.0-0.7] | 0.2 [0.0-0.3] |
| MIP-1α | 1,460.1 [1,062.1-1,631.8] | 1,934.3 [1,364.8-2,104.9] | 350.3 [247.0-484.6] |  | 1,666.3 [1,018.3-1,896.4] | 1,814.1 [1,193.2-2,410.0] | 223.6 [106.0-354.5] |
| IL-36RA | 168.8 [121.8-205.0] | 153.2 [105.5-196.0] | (-)12.0 [(-)16.4-(-)5.8] |  | 158.7 [128.0-167.2] | 141.3 [111.7-164.1] | (-)4.9 [(-)16.3-1.9] |
| IP-10 | 874.1 [758.3-995.4] | 1,228.7 [1,051.4-1,695.7] | 433.3 [180.4-614.1] |  | 641.2 [545.4-836.0] | 613.8 [600.7-802.4] | (-)25.2 [(-)39.8-16.0] |
| MCP-1 | 921.1 [905.6-1,010.8] | 949.9 [911.4-1,056.5] | 17.9 [0.1-38.5] |  | 924.8 [847.8-936.6] | 898.1 [839.4-950.2] | (-)3.9 [(-)10.8-44.7] |
| MIP-1β | 0.0 [0.0-0.0] | 0.0 [0.0-0.0] | 0.0 [0.0-0.0] |  | 0.0 [0.0-0.0] | 0.0 [0.0-0.0] | 0.0 [0.0-0.0] |
| TNF-α | 1,763.6 [849.8-3,744.7] | 1,639.0 [838.5-3,346.4] | (-)48.2 [(-)128.5-(-)39.7] |  | 668.2 [325.1-2,070.5] | 677.5 [334.7-2,225.0] | 9.2 [(-)43.3-26.4] |
| VEGF-A | 298.1 [222.0-375.6] | 222.0 [172.4-318.9] | (-)44.2 [(-)56.8-(-)33.5] |  | 332.6 [312.2-417.8] | 333.3 [289.8-392.6] | (-)23.8 [(-)33.8-(-)22.44] |

^a^ Concentrations (pg/mL) measured with the MILLIPLEX® Bovine Cytokine/Chemokine Magnetic Bead Panel 1.

^b^Δ-cytokine was calculated as the difference between the cytokine level under PPDb stimulation and its paired PBS baseline.
